# Chromatin modifications and DNA repair: beyond double-strand breaks

**DOI:** 10.3389/fgene.2014.00296

**Published:** 2014-09-05

**Authors:** Nealia C. M. House, Melissa R. Koch, Catherine H. Freudenreich

**Affiliations:** ^1^Department of Biology, Tufts UniversityMedford, MA, USA; ^2^Program in Genetics, Sackler School of Graduate Biomedical Sciences, Tufts UniversityBoston, MA, USA

**Keywords:** histone modification, chromatin remodeler, DNA structure, sister chromatid recombination, gap repair, stalled replication fork, excision repair, mismatch repair

## Abstract

DNA repair must take place in the context of chromatin, and chromatin modifications and DNA repair are intimately linked. The study of double-strand break repair has revealed numerous histone modifications that occur after induction of a DSB, and modification of the repair factors themselves can also occur. In some cases the function of the modification is at least partially understood, but in many cases it is not yet clear. Although DSB repair is a crucial activity for cell survival, DSBs account for only a small percentage of the DNA lesions that occur over the lifetime of a cell. Repair of single-strand gaps, nicks, stalled forks, alternative DNA structures, and base lesions must also occur in a chromatin context. There is increasing evidence that these repair pathways are also regulated by histone modifications and chromatin remodeling. In this review, we will summarize the current state of knowledge of chromatin modifications that occur during non-DSB repair, highlighting similarities and differences to DSB repair as well as remaining questions.

## INTRODUCTION

Assaults to the genome are common throughout the lifetime of a cell and DNA damage can occur by endogenous factors, such as reactive oxygen species, base mismatches, and alternative (non-B form) DNA structures, or exogenous factors, such as ultraviolet (UV) radiation and environmental toxins. At the occurrence of a DNA lesion, the cell will initiate repair to protect the integrity of the genetic material. As the genome is condensed into chromatin, repair must work within the context of the chromatin structure to access and repair the damaged DNA.

One mechanism to alter chromatin structure is to modify histone residues by the addition of chemical groups such as a phosphate, acetyl, or one or more methyl groups. Small peptides such as ubiquitin and SUMO can also be added to lysine residues. These histone modifications can change nucleosome-DNA or nucleosome–nucleosome interactions to either open or condense the chromatin structure. For example, acetylation of histone H4 at lysine 16 (H4K16ac) reduces the interaction between the H4 tail and the H2A acidic pocket, inhibiting higher-order nucleosomal folding and resulting in a more open chromatin confirmation (Shogren-Knaak et al., 2006; Robinson et al., 2008). Alternatively, histone modifications that occur upon DNA damage can alter the interaction of non-histone proteins with chromatin to facilitate direct recruitment of repair factors and contribute to checkpoint initiation and termination (Humpal et al., 2009). ATP-dependent chromatin remodelers are also actively altering the chromatin landscape to promote repair (Seeber et al., 2013). Remodelers can slide nucleosomes, evict whole or partial nucleosomes, or alter the interaction between nucleosomes and DNA (Seeber et al., 2013). Still, in many cases, the details of how histone modifications and chromatin remodeling are affecting the formation, or progression of repair intermediates is not well understood. These intermediates include replication fork stabilization, strand resection, gap filling, and strand invasion or extension. The efficiency of formation or resolution of repair intermediates could ultimately dictate repair-pathway choice.

**FIGURE 1:**
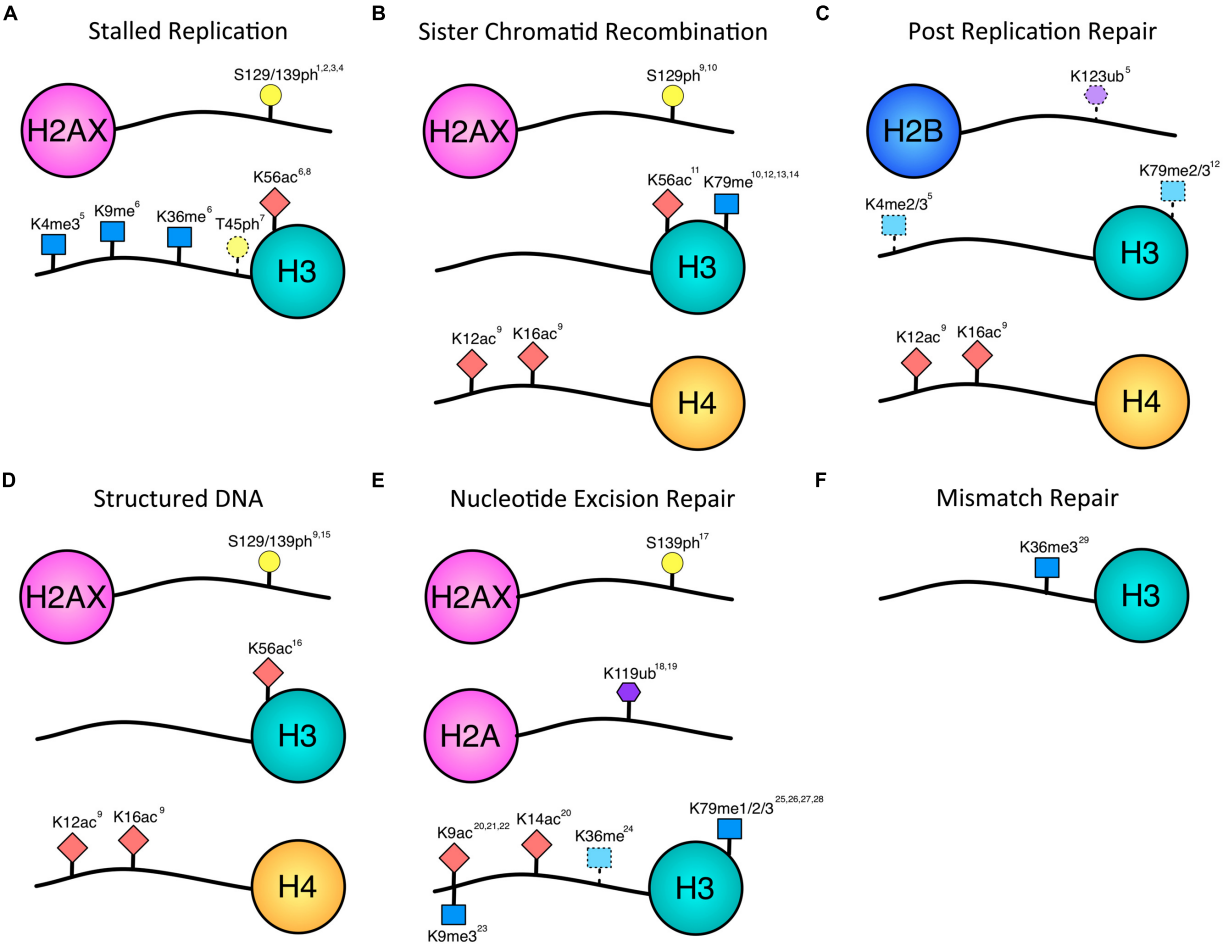
**Histone modifications associated with repair of single-stranded lesions.** Histone phosphorylation (yellow circle), acetylation (red diamond), methylation (blue square), and ubiquitination (purple hexagon) have all been implicated in repair outside of DSBs. Numbering of modified residues is according to the organism in which the modification was identified in the referenced work. Dotted lines indicate uncertainty of pathway association. **(A)** Histone modifications documented to occur in response to stalled replication forks. There is overlap with modifications associated with DSBs, and future data may provide further distinction between stalled and collapsed forks. **(B)** Modifications associated with sister chromatid recombination in response to gaps or DSBs. While the H3 modifications have been implicated in DSB SCR, the H2A and H4 modifications have been associated with the fidelity of gap-induced SCR. **(C)** H4-K12ac, and H4-K16ac have been implicated in error-free PRR. H2B-K123ub, H3-K4me, H3-K79me are dependent on Rad6, which is required for PRR. However, they have not yet been shown to be necessary for PRR and may contribute to other homology-mediate repair events. **(D)** Histone modifications associated with structured DNA. These modifications also impact the fidelity with which the DNA is repaired. **(E)** Histone modifications occurring during nucleotide excision repair. A decrease in H3-K9me is indicated by the downward facing blue square. H3-K36 methylation may be associated with transcription and/or TCR. **(F)** The only histone modification shown to be necessary so far for mismatch repair is H3K36 methylation. ^1^Cobb et al. (2005), ^2^Szilard et al. (2010), ^3^Ward and Chen (2001), ^4^Sirbu et al. (2011), ^5^Faucher and Wellinger (2010), ^6^Kim et al. (2008), ^7^Baker et al. (2010), ^8^Wurtele et al. (2012), ^9^House et al. (2014), ^10^Conde et al. (2009), ^11^Munoz-Galvan et al. (2013), ^12^Game et al. (2006), ^13^Toh et al. (2006), ^14^Grenon et al. (2007), ^15^Entezam and Usdin (2008), ^16^Yang and Freudenreich (2010), ^17^O’Driscoll et al. (2003), ^18^Kapetanaki et al. (2006), ^19^Bergink et al. (2006), ^20^Yu et al. (2005), ^21^Guo et al. (2011), ^22^Rubbi and Milner (2003), ^23^Palomera-Sanchez et al. (2010), ^24^Malik et al. (2010), ^25^Evans et al. (2008), ^26^Bostelman et al. (2007), ^27^Chaudhuri et al. (2009), ^28^Tatum and Li (2011), ^29^Li et al. (2013).

DNA double-strand breaks (DSBs) are considered to be the most lethal type of DNA damage and the chromatin factors mediating repair of these lesions have been extensively studied. However, DSBs are rare, and more common threats to the genome include single-strand DNA gaps, nicks, base lesions, stalled replication forks, and non-canonical DNA topology that can interfere with replication and repair. The chromatin modifications that are occurring during these other types of DNA repair pathways remain less well-characterized than DSB repair because of the technical difficulty associated with studying a site-specific, non-DSB lesion compared to robust DSB-inducing systems. However, studies have started to elucidate the contribution of histone modifications to non-DSB lesions (**Figure 1**). This review will focus on the chromatin modifications known to date to contribute to repair of single-strand DNA gaps, stalled forks, DNA structures, base lesions and mismatches, and will compare and contrast these marks to those known to occur during DSB repair.

## CHROMATIN MODIFICATIONS AND REMODELING DURING DSB REPAIR

At the occurrence of a DSB, the MRN (MRX in yeast) complex binds the broken DNA ends, which recruits the PIKK kinases, ATR/ATM (Mec1/Tel1 in yeast); alternatively, the broken ends are bound by the Ku70/Ku80 heterodimer, which recruits DNA-PK (Gottlieb and Jackson, 1993; Mahaney et al., 2009). These kinases phosphorylate H2AX, thus forming γH2AX and initiating the chromatin response to DNA damage (Rogakou et al., 1998; Downs et al., 2004; Ataian and Krebs, 2006; Bao, 2011). The histone modifications documented to occur during DSB repair will be briefly summarized here in order to provide context for comparison to other repair pathways. For recent, more detailed reviews, see Chubb and Rea (2010), Zhu and Wani (2010), Bao (2011), Greenberg (2011), Miller and Jackson (2012), Seeber et al. (2013), Price and D’Andrea (2013), and Tsabar and Haber (2013).

### γH2AX AT DSBs

γH2AX (H2AX-S139ph in mammals; H2A-S129ph in yeast) is the most well-documented histone modification in response to a DNA DSB and occurs within minutes of break induction (Rogakou et al., 1998; Shroff et al., 2004; Stiff et al., 2004, 2006). The γH2AX domains are established by a positive feedback loop whereby γH2AX recruits the mammalian repair mediator MDC1, which in turn recruits additional MRN that will stimulate further phosphorylation of H2AX by ATM (Uziel et al., 2003; Lukas et al., 2004; Lee and Paull, 2005; Stucki et al., 2005; Lou et al., 2006). This MDC1-ATM pathway to amplify the γH2AX signal increases the density of γH2AX proximal to the break site; however, subsequent spreading of γH2AX to create large domains is dependent on the action of ATM, but not MDC1 (Savic et al., 2009). Earlier data in yeast indicated that the γH2AX modification is necessary for the recruitment of chromatin modifying enzymes, including INO80 and SWR1 remodelers (Downs et al., 2004; Morrison et al., 2004; van Attikum et al., 2004), which can alter the chromatin structure to allow access by other repair proteins, such as 53BP1, Rad51, and BRCA1 (Paull et al., 2000; Celeste et al., 2003a,b; Murr et al., 2006; Shi and Oberdoerffer, 2012; Scully and Xie, 2013). However, recent work in yeast demonstrated that the γH2AX modification is dispensable for recruitment of the chromatin modifying enzymes INO80, SWR-C, NuA4, SWI/SNF, or RSC to DSBs during homologous recombination in G2 cells (Bennett et al., 2013), suggesting that the repair proteins are not necessarily directly recruited by an interaction with γH2AX. Instead, recruitment of chromatin modifiers and remodelers in G2 is tightly coupled to homologous recombination, and the Rad51 filament itself may play a role (Bennett et al., 2013). In any case, γH2AX modification is an early step in a cascade of chromatin modifications, including nucleosome remodeling and other post-translational histone modifications, which allows for subsequent recruitment and retention of repair factors. Although many histone modifications that contribute to DSB repair have been identified, the order of events is only partially understood (Bao, 2011).

In addition to γH2AX, other histone modifications are required for efficient repair of DSBs, including acetylation, methylation, and ubiquitination of lysine residues. The modification of amino acid residues can be influenced by existing histone modifications. For example, H4-S1 phosphorylation after DNA damage is required for H4 N-tail lysine deacetylation (Cheung et al., 2005; Utley et al., 2005), and H2B ubiquitination is required for H3 methylation (Game and Chernikova, 2009; discussed below). This presents the interesting possibility that ordered progression of modifications could allow for regulation of repair events or be important in promoting proper repair.

### HISTONE METHYLATION

Defective methylation of H3-K79 and H3-K36 results in ionizing radiation (IR) sensitivity in yeast cells (Game et al., 2005, 2006; Grenon et al., 2007; Game and Chernikova, 2009). In mammalian cells, H3-K79 methylation, along with H4-K20 dimethylation, are recognized by 53BP1 in relaxed chromatin at the DSB (Hartlerode et al., 2012; Wakeman et al., 2012; Hsiao and Mizzen, 2013). H3-K9me3, on the other hand, stimulates TIP60 histone acetyltransferase (HAT) activity at the break site, resulting in acetylation of both histones and ATM, the latter activating the kinase to further stimulate γH2AX formation (Ikura et al., 2000; Murr et al., 2006; Xu et al., 2010, 2012; Bao, 2011; Xu and Price, 2011).

### HISTONE UBIQUITINATION

Histone ubiquitination has been implicated in several steps of DSB repair (Bao, 2011). H2AX-K119 ubiquitination is induced upon IR treatment (Xie et al., 2010) and is required for histone turnover at the site of damage (Ikura et al., 2007). H2A/H2A.X ubiquitination by RNF8 and RNF168 is also required for accumulation and retention of 53BP1 and BRCA1 at the break (Huen et al., 2007; Kolas et al., 2007; Mailand et al., 2007; Doil et al., 2009). H3 and H4 ubiquitination have also been shown to facilitate the recruitment of repair factors to a DSB, and in mammalian cells monoubiquitination of H2B-K120 is required for recruitment of both HR and NHEJ repair factors and may contribute to chromatin decompaction to promote repair (Wang et al., 2006; Moyal et al., 2011). In yeast, ubiquitination of H2B-K123 is a prerequisite for H3-K4 and H3-K79 methylation and is necessary for Rad53 phosphorylation in response to DNA damage (Giannattasio et al., 2005).

### HISTONE ACETYLATION AND DEACETYLATION

Histone acetylation flanking a DSB is required for repair and cellular survival after DNA damage in both yeast and mammalian cells (Bird et al., 2002; Downs et al., 2004; Tamburini and Tyler, 2005; Murr et al., 2006; Xu et al., 2010). In yeast, histone lysine residues are acetylated at DSB sites by Gnc5, an H3-specific HAT recruited by γH2AX (Lee et al., 2010); in mammalian cells, histones are acetylated by TIP60, the NuA4 complex HAT that is recruited to a DSB by a physical interaction with the MRN complex (Chailleux et al., 2010). An additional mammalian HAT, MOF, acetylates histone H4-K16 and this modification is required for the recruitment of repair factors to an irradiation-induced break site, including MDC1, 53BP1, and Brca1 (Li et al., 2010; Krishnan et al., 2011).

As histone modifications are required to alter the chromatin environment to facilitate repair, additional modifications are required to reset the chromatin state once repair is complete. Histone deacetylases (HDACs) are recruited to remove histone acetyl marks and restore the chromatin structure in yeast (Tamburini and Tyler, 2005). However, HDACs may also play a more direct role in the repair process, as in mammalian cells HDACs are recruited to a DSB early in the repair process (Bao, 2011; Xu and Price, 2011). In mammalian cells, the HDAC SIRT1 is recruited to an I-SceI DSB (Oberdoerffer et al., 2008), and the HDAC complex NuRD, which includes HDAC1 and HDAC2, is recruited to a microirradiation-induced DSB to deacetylate H3-K56 (Miller et al., 2010), a histone modification that promotes nucleosome assembly during replication and repair (Chen et al., 2008; Li et al., 2008).

### CHROMATIN REMODELERS

Chromatin remodelers have also been shown to play an important role in DSB repair. Interestingly, the NuRD HDAC complex contains a chromatin remodeler subunit (MTA1 or 2), and the NuA4 HAT complex contains the chromatin remodeler p400 (Xu and Price, 2011; Price and D’Andrea, 2013), intimately linking the role of histone modifications and chromatin remodeling. p400 (SWR1 in yeast) has recently been shown to catalyze the exchange of the H2A variant H2A.Z onto the chromatin at DSBs, which leads to a more open chromatin structure and promotes further histone modifications at the site of damage (Xu et al., 2012). Once at the site of damage, SWR1 stimulates the exchange of H2A.Z onto the chromatin and this exchange is promoted by both H2A and H4 acetylation (Altaf et al., 2010). In yeast, the direction of exchange (H2AX for H2A.Z or H2A.Z for H2AX) is influenced by H3-K56 acetylation state: SWR-C preferentially removes H2A.Z from the nucleosome when H3 is acetylated at K56 (H3-K56Q acetyl-mimic mutant), and thus the specific catalytic activity of the SWR-C remodeler is determined by histone modification state to promote turnover of histone variants (Watanabe et al., 2013). SWR1 also facilitates Ku80 binding at the break, thereby promoting NHEJ (van Attikum et al., 2007; Bao, 2011).

Chromatin remodelers appear to play a key role during repair, but the exact function of many remodelers remains unknown. What DNA topological substrate is specifying remodeler recruitment or action and how histone modifications contribute to this process or remodeler function remain to be elucidated. Chromatin remodelers may be required during repair to open the damaged DNA to other repair proteins; alternatively, remodelers could be important to downregulate transcription in the vicinity of the break to limit collisions between the repair machinery and transcription machinery, allowing repair to progress properly (Kruhlak et al., 2007; Shanbhag et al., 2010). More generally, a transient repressive chromatin state may be important for stabilization of the chromatin fiber for efficient repair, as was a proposed role for H3-K9me3 at a DSB (Ayrapetov et al., 2014). In addition to physically altering the chromatin to facilitate proper access to the DNA template, remodelers could be more directly involved in the subsequent cascade of damage signaling by directly interacting with other repair factors, perhaps acting as recruitment platforms or mediators.

## CHROMATIN MODIFICATIONS ASSOCIATED WITH STALLED REPLICATION FORKS

Stalled replication forks can be protective to genomic integrity, given that the stall can avoid replication through damaged DNA and signal the location of DNA damage to be repaired. However, if the damage is not repaired or bypassed, or if a single-strand break is in the template, the stalled fork can collapse, leading to a DSB. For example, low doses of aphidicolin can induce replication stress that will stall forks and leave single-strand gaps, eventually resulting in DSBs (Freudenreich, 2007). Interestingly, aphidicolin treatment during S phase induces γH2AX-dependent 53BP1 foci in the next G_1_ phase, indicating that a fork stall not resolved by mitosis can lead to a DSB in the next cell cycle (Harrigan et al., 2011; Lukas et al., 2011).

To prevent DSB formation, damage tolerance pathways can be invoked, leaving the damage to be resolved through post-replication repair (PRR). Error-prone PRR occurs by recruitment of a translesion synthesis (TLS) polymerase that can bypass the lesion. Alternatively, a template switch involving sister chromatid annealing can allow the polymerase to copy the homologous sequence information from the sister chromatid and continue replication, or sister chromatid recombination (SCR) can be used to repair a gap left after fork passage. Thus, since fork stalling can lead to TLS, template switching, SCR, or a DSB, it can be experimentally difficult to distinguish the chromatin modifications that are specific to the initial fork stall or to each subsequent repair pathway. Stalling replication forks with low levels of hydroxyurea (HU) or inducing site-specific stalls with DNA-bound proteins or known fork-stalling DNA sequences, such as CGG repeats, can be effective strategies to uncover chromatin modifications associated with stalled replication forks. In addition, co-localization experiments using ChIP or isolation of proteins on nascent DNA (iPOND, Sirbu et al., 2012; see below) have been productive in linking replication fork-associated factors with chromatin-associated factors. This section will focus on the histone modifications and chromatin remodelers known to-date to be associated with stalled replication forks.

Stalled replication forks are marked in the chromatin as DNA damage, as γH2AX domains form at stalled replication forks (**Figure 1A**). In yeast, phosphorylated H2A (γH2A) was found to co-localize with HU stalled forks and Pol 𝜀 by ChIP, and this event was dependent on the Mec1 but not the Tel1 kinase, distinguishing the modification from γH2A at a DSB which can be phosphorylated by both Mec1 and Tel1 (Cobb et al., 2005). Indeed, genome-wide mapping of γH2A-rich loci using ChIP technology revealed that γH2A is enriched at sites of natural replication fork stalling, including the rDNA locus, tRNA genes, LTRs, telomeres, and DNA replication origins (Szilard et al., 2010). Interestingly, the average size of the γH2A domain at these natural pause sites was 1255 bp, in contrast to the 50 kb domain detected at an HO endonuclease-induced DSB in yeast cells. Functionally, H2A modification is required to promote replication fork progression, as measured by total DNA content after release from G_1_ in *mec1-ts* mutants, and prevent DSB formation, as measured by pulsed-field gel electrophoresis (Cha and Kleckner, 2002).

In mammalian cells, γH2AX is induced when DNA replication is inhibited by HU, forming foci that co-localize with PCNA in S phase cells, and this response is also dependent on ATR but not ATM (Ward and Chen, 2001). Using iPOND technology to monitor protein dynamics at sites of newly synthesized DNA in live mammalian cells, γH2AX was detected at a stalled replication fork within 10 min after HU addition, becoming maximal at 30 min (Sirbu et al., 2011). These early time points were not accompanied by markers of DSBs such as Mre11, DNA-PK, or Ku70/Ku80 which appeared at later time points 2–4 h after HU addition, indicating that the early γH2AX is not marking collapsed forks or DSBs. The γH2AX domain spread from the site of fork stalling over time, reaching tens of thousands of base pairs by 1 h. Again, initial γH2AX formation at an HU-stalled replication fork was ATR-dependent; but maintenance of the γH2AX domain at later time points was ATM-dependent, likely occurring once the persistently stalled fork had collapsed into a DSB (Sirbu et al., 2011).

While both DSBs and stalled forks are marked by an initial γH2AX histone modification, subsequent chromatin modifications dependent on either ATM or ATR could produce chromatin environments specific to the lesion type, directing repair to the appropriate pathway or influencing the repair process itself. The histone modifications important for turning off the DNA damage response at a stalled fork may also be different than at a DSB. To turn off the DSB-induced checkpoint, mammalian serine/threonine phosphatase complexes PP2A and PP4 and the yeast PP4C ortholog Pph3 dephosphorylate γH2AX, leading to inactivation of Rad53 (Chowdhury et al., 2005; Keogh et al., 2006; Nakada et al., 2008). However, another phosphatase, PP1 (Glc7), has been shown to dephosphorylate γH2AX and contribute to Rad53 inactivation and replication fork restart after HU treatment (Bazzi et al., 2010). Of note, PP4 in mammalian cells appears to be especially important for resolution of DNA damage that occurs during replication, specifically dephosphorylating ATR (but not ATM)-modified γH2AX (Chowdhury et al., 2008).

Although much research has focused on γH2AX at stalled replication forks, other histone modifications are likely occurring to influence replication fork recovery or repair (**Figure 1A**). One such modification is phosphorylation of H3-T45 in yeast, a modification observed in response to prolonged replication stress in HU treated cells that is independent of the Mec1 and Tel1 kinases and is instead regulated by the Cdc7-Dbf4 kinase complex (Baker et al., 2010). The authors conclude that this modification is specific to replication stress, as treatment with DNA alkylating agent MMS did not increase H3-T45 phosphorylation. Further, the H3-T45A mutant was not sensitive to MMS, but was sensitive to HU and CPT, a topoisomerase I inhibitor, as measured by cell survival in a spot assay (Baker et al., 2010). However, prolonged exposure to HU and CPT will lead to DSB formation and therefore this modification could mark DSBs, although in a Mec1/Tel1-independent manner.

In addition to histone phosphorylation, histone acetylation, methylation, and ubiquitination likely play a role in signaling replication stress (**Figure 1A**). In human T-cell lymphoma cells, HDAC3 is localized to replication forks by iPOND, linking changing acetylation state with newly synthesized DNA (Wells et al., 2013). Further, HDAC3 inhibition resulted in decreased replication fork velocity and increased apoptosis that was associated with increased DNA damage and an S phase defect (Wells et al., 2013). In budding yeast, H3-K56 acetylation is required to complete replication in the presence of lesions caused by MMS (Wurtele et al., 2012). In fission yeast, the absence of Clr4 and Set2, the methyltranferases for H3-K9 and H3-K36, respectively, leads to a decrease in HU-induced phosphorylation of Cdc2 and Mik1, downstream actors in the Rad3 (human ATM) checkpoint pathway. Therefore, the authors conclude that the HU replication stress checkpoint requires H3 methylation by Clr4 and Set2 (Kim et al., 2008). H3-K4 trimethylation may also contribute to repair of S phase damage in *S. cerevisiae,* as the absence of Set1, the HMT responsible for H3-K4 trimethylation, leads to an S phase progression defect, in addition to the role of Set1 in NHEJ (Faucher and Wellinger, 2010). Histone H3-K4 and K79 methylation, regulated by H2B-123 ubiquitination, may also play a role in PRR, which would be initiated after a replication fork stalling event (discussed in next section).

Chromatin remodeling is also important in resolving the damage at a stalled replication fork (**Table 1**). INO80 is implicated in recovery from stalled replication in both budding yeast and mammalian cells. In mammalian cells, *ino80* mutants are HU sensitive, display defective S phase progression, and are defective in recovery from replication stress (Hur et al., 2010; Min et al., 2013; Vassileva et al., 2014). In yeast, Ino80 is enriched at stalled replication forks, as detected by ChIP (Papamichos-Chronakis and Peterson, 2008; Shimada et al., 2008). In addition, recovery from replication fork stalling after HU treatment is impaired in an *ino80* mutant, resulting in DSBs (Shimada et al., 2008), and Ino80 promotes replication restart after MMS treatment (Falbo et al., 2009). In the absence of both an intact INO80 complex and the chromatin remodeler Isw2, recovery from the S phase checkpoint response is defective (Au et al., 2011). The chromatin remodeler RSC2 may also play a role in recovery from stalled replication or be involved in PRR. In *S. cerevisiae,* RSC2 is found near replication forks by ChIP, and PCNA ubiqutination is significantly decreased in a *rsc2Δ* mutant after MMS, UV, and HU treatments (Niimi et al., 2012). Similarly, depletion of the human homolog BAF180 of the PBAF complex led to a decrease in fork progression by IdU incorporation (DNA fiber) analysis and decreased chromatin bound unmodified and ubiquitin-modified PCNA and Rad18 (Niimi et al., 2012). Other remodelers are found at replicating forks irrespective of a stall, but may also play a role at stalled forks (Vincent et al., 2008; Au et al., 2011; Bhaskara et al., 2013).

**Table 1:** Chromatin remodelers associated with repair pathways outside of DSB repair.

Repair pathway	Remodeling complex	Implicated subunit	System	Reference
**Stalled Replication**	INO80	Ino80	Yeast	Papamichos-Chronakis and Peterson (2008), Shimada et al. (2008), Falbo et al. (2009)
			Human	Hur et al. (2010), Vassileva et al. (2014)
			Mouse	Min et al. (2013)
	ISW2	Isw2	Yeast	Vincent et al. (2008), Au et al. (2011)
	RSC	Rsc2	Yeast	Niimi et al. (2012)
	PBAF(RSC ortholog)	BAF180	Human cells	Niimi et al. (2012)

**SCR**	RSC	Rsc1	Yeast	House et al. (2014)
		Rsc2	Yeast	Baetz et al. (2004), Oum et al. (2011), House et al. (2014)
		Rsc7	Yeast	Oum et al. (2011)

**PRR**	RSC	Rsc2	Yeast	Niimi et al. (2012), House et al. (2014)

**Structured DNA**	RSC	Rsc1	Yeast	House et al. (2014)
		Rsc2	Yeast	House et al. (2014)
	SWR1	Bdf1	Yeast	House et al. (2014)

**GGR**	SWI/SNF	Snf2	Yeast	Yu et al. (2005)
		Snf5	Yeast	Gong et al. (2006)
			Human cells	Ray et al. (2009), Zhao et al. (2009), Zhang et al. (2009)
		Snf6	Yeast	Gong et al. (2006)
		BRG1	Human cells	Zhao et al. (2009), Zhang et al. (2009)
	SWI/SNF-like	Rad16	Yeast	Ramsey et al. (2004), Yu et al. (2011)
		ALC1	Human cells	Pines et al. (2012)
	INO80	Ino80	Yeast	Sarkar et al. (2010)
		Ino80, Arp5	Human cells	Jiang et al. (2010)
	ISWI	ACF	*in vitro* (*Drosophila*)	Ura et al. (2001)

**TCR**	SWI/SNF-like	CSB	*in vitro* (Human)	Citterio et al. (2000)
		Rad26	Yeast	Gregory and Sweder (2001)
	ISW1	SMARCA5/SNF2H, WSTF, ACF	Human cells	Aydin et al. (2014)

**BER**	ISW1, ISW2	Isw1, Isw2	*in vitro* (yeast)	Nakanishi et al. (2007)
	RSC	Sth1	Yeast	Czaja et al. (2014)
	SWI/SNF	Complex	*in vitro* (yeast)	Menoni et al. (2007)

It is likely that additional histone modifications are associated with recovery from stalled replication forks, but they remain to be identified. Histone modifications could influence several steps of recovery from stalled replication, including marking the location of a stalled fork, recruitment of replication restart factors or replication bypass factors (including translesion synthesis polymerases), establishing sister chromatid cohesion for homology-mediated PRR, and finally the recruitment of chromatin modifying enzymes to reset the chromatin structure.

## CHROMATIN MODIFICATIONS IN RESPONSE TO SINGLE-STRAND GAPS REPAIRED BY SISTER CHROMATID RECOMBINATION OR TEMPLATE SWITCHING

More common than DSBs are single strand DNA lesions that can occur during replication and repair. Single strand gaps that occur during replication will activate the DNA damage checkpoint (Branzei and Foiani, 2008). These gaps activate the kinase ATR, not ATM, and the intensity of the checkpoint response increases with increasing gap length, as monitored by Chk1 phosphorylation (MacDougall et al., 2007). To prevent gaps from becoming DSBs, Rad6-Rad18 dependent damage tolerant replication can be invoked to allow replication to continue, followed by subsequent repair of the template-strand lesion in a process that has been termed PRR. Single-stranded gaps that occur during replication must be resolved before the following S phase to prevent the formation of DSBs. Base damage, for example by alkylating agents such as MMS, result in unreplicated gaps left after fork passage (Hashimoto et al., 2010). It is probable that repair of nicks and gaps will have overlapping histone modifications with DSB repair, particularly if the lesion induces a checkpoint response. However, it is also likely that different combinations of histone modifications will distinguish nick and gap repair pathways from repair of a DSB.

### POST-REPLICATION REPAIR

Post-replication repair can be divided into two Rad6-dependent, damage tolerant pathways: error-prone TLS and error-free PRR. TLS is initiated by Rad6-Rad18 monoubiquitination of PCNA and allows replication past a lesion by employing low-fidelity translesion polymerases with large active sites that can accommodate bulky lesions. Rad6 is the E2 ubiquitin-conjugating enzyme that cooperates with the E3 ubiquitin ligase Rad18 to modify PCNA to initiate PRR. However, with the E3 ubiquitin ligase Bre1, Rad6 also plays a role in regulating histone H2B-K123 ubiquitination (Briggs et al., 2002; Dover et al., 2002; Ng et al., 2002; Game and Chernikova, 2009). H2B-K123 ubiquitination promotes H3-K4 and H3-K79 di- and tri-methylation by Set1 and Dot1, respectively (Briggs et al., 2001; Miller et al., 2001; Shahbazian et al., 2005; Fuchs et al., 2009; Nakanishi et al., 2009; Takahashi et al., 2009). Given the regulation by Rad6, H2B-K123 ubiquitination and H3-K4 and H3-K79 methylation may play a role in PRR (**Figure 1C**). To date, no histone modifications are identified to contribute specifically to the TLS branch of PRR, but the HAT Gcn5 is required for transcription of the TLS polymerase η (Kikuchi et al., 2012).

Error-free PRR requires polyubiquitination of PCNA by Ubc13-Mms2 and Rad5, which initiates a template switch to bypass the template strand lesion and copy from the newly synthesized sister chromatid. The recombination event during error-free PRR further requires the action of Rad52 epistasis group members (Boiteux and Jinks-Robertson, 2013). Thus, a post-replication template switch is mechanistically very similar to gap-induced sister chromatid recombination, and may be marked by similar or identical histone modifications. Until recently, no particular histone modifications had been attributed to error-free PRR or gap-induced SCR. However, our group has recently found that acetylation of histone H4 by the HAT Esa1 of the NuA4 complex is needed for stability of CAG repeats in a Rad5-dependent manner (**Figure 1C**). Furthermore, the HAT activity of NuA4 is required for gap-induced SCR. The most important modifications are acetylation of H4-K12 and H4-K16, known targets of Esa1 (**Figure 1B**). Additionally, H4-K16 acetylation at the CAG repeat peaks during S phase, but then disappears, presumably once repair is complete. A dynamic nature to the histone acetylation appears to be important in maintaining genomic stability, as both HAT and HDAC mutants displayed an increased frequency of CAG repeat expansions. If histone acetylation was primarily acting to disrupt higher order chromatin compaction to open the chromatin structure (Murr et al., 2006; Shogren-Knaak et al., 2006), the HDAC mutant would have rescued genomic stability by allowing constant decompaction of the chromatin. As this was not the case, the requirement for dynamic histone H4 acetylation argues for a model in which the modification is directly affecting recruitment or turnover of repair factors to facilitate PRR of gaps via SCR.

### SISTER CHROMATID RECOMBINATION

Sister chromatid recombination is a homology-mediated event that contributes to both DSB repair when a sister chromatid is available as well as post-replication single strand gap repair. Chromatin modifications associated with SCR have been identified mostly within the context of a DSB, but since the physical recombination event in DSB and PRR will be similar, it is reasonable to expect that some histone modifications will affect both repair pathways. One potential example of this is H3-K56 acetylation. Not only does H3-K56 acetylation respond to replication fork damage (Wurtele et al., 2012), but it also works with Rad52 to promote SCR during repair of a DSB (Munoz-Galvan et al., 2013; **Figures 1A,B**). H3-K56 acetylation was also shown to be important in preventing CAG repeat fragility and contractions during both replication and Rad52-dependent repair events (Yang and Freudenreich, 2010; **Figure 1D**). In human cells, TIP60-dependent H4 acetylation has been shown to promote BRCA1-dependent HR (Tang et al., 2013), and depletion of the H4K16-specific HAT MOF leads to a decrease in DSB-induced HR and sister-chromatid exchanges (Li et al., 2010; Sharma et al., 2010), suggesting that H4 acetylation is important in facilitating homology-dependent recombination events between sister chromatids.

Sister chromatid cohesion is necessary for proper alignment of homologous sequences during SCR. Contributing to this process is the RSC complex, which is required to recruit cohesin to chromosomes (Baetz et al., 2004), and also to recruit the cohesin subunits Smc1 and Scc1 to a DSB (Oum et al., 2011). These results link chromatin remodeling to cohesin loading during recombination. Lending support to a role for RSC in SCR is that *rsc2* or *rsc7* deletions confer sensitivity to MMS during G_2_ but not G_1_, indicating RSC is most important after synthesis when the sister chromatid would be available as a template for repair. Indeed, the *rsc7* mutant has a decrease in spontaneous sister chromatid exchange (Oum et al., 2011), and we found that both *rsc1* and *rsc2* mutants were defective in spontaneous SCR (House et al., 2014; **Table 1**). Interestingly however, only Rsc2 is able to suppress an MMS-induced increase in SCR, implicating the Rsc2 sub-complex specifically in gap-induced SCR (**Table 1**; House et al., 2014). Additionally, the Rsc2 sub-complex is detected by ChIP at an unstable CAG repeat coincident with H4K16ac, suggesting a possible recruitment mechanism for this remodeler during gap-induced repair from the sister chromatid (House et al., 2014). Both efficient γH2AX modification at a break site (Kent et al., 2007) and MRX recruitment to a DSB (Shim et al., 2007) are dependent on RSC. Therefore, this chromatin remodeling complex may be a common component of HR repair induced by either a DSB or gap that links the initial damage event to the subsequent chromatin response.

In addition to histone acetylation, both histone methylation and phosphorylation are required for proper SCR (**Figure 1B**). In yeast, the histone methyltransferase Dot1 has specificity for the H3-K79 residue and is required for DSB break repair (Game et al., 2006; Game and Chernikova, 2009). In the absence of Dot1, cells lose IR-induced Rad9 foci in G_2_, suggesting a role for this modification in recruitment of Rad9 specifically when a sister chromatid is present (Toh et al., 2006; Grenon et al., 2007). Further, using a physical assay to probe for recombination intermediates and monitor unequal exchange of sister chromatids upon replication of a DSB lesion, Conde et al. (2009) found that the unphosphorylatable H2A-S129A mutant and the *dot1Δ* mutant are defective in SCR and are contributing to repair by promoting sister chromatid cohesion (Conde et al., 2009).

As the chromatin modifications and remodeling required to promote single-strand gap repair, PRR, and SCR continue to be defined, it is probable that more specific combinations of histone modifications will be revealed to be distinct from those required for DSB repair. Histone modifications could contribute to altering the chromatin environment to promote recombination, either directly by changing charge interactions between nucleosomes and the DNA, or indirectly through repair factor and chromatin remodeler recruitment. Specific histone modifications and remodelers may also contribute to the overall fidelity of repair, as illustrated by those that are needed to promote SCR as well as prevent CAG repeat instability (**Figures 1B,D**).

## THE CHROMATIN RESPONSE TO STRUCTURE-FORMING DNA

Non-canonical DNA topology can lead to DNA lesions and must be resolved to prevent the loss of genomic material. Inverted repeats, some direct repeats, and homopyrimidine-homopurine runs can form stable secondary structures that impede DNA processing events, such as replication and repair, causing DNA damage and genome instability (Freudenreich, 2007; Voineagu et al., 2009a; Kim and Mirkin, 2013). Since DNA structures can cause all of the types of damage covered above, including DSBs, stalled forks, and single-strand DNA gaps or nicks, the modifications associated with structure-forming DNA will overlap with those found at these lesions. Indeed, structure-forming CGG/CCG and CAG/CTG triplet repeats induce replication fork stalling and chromosome fragility when they reach a length of 45–70 repeats (Samadashwily et al., 1997; Freudenreich et al., 1998; Balakumaran et al., 2000; Jankowski et al., 2000; Callahan et al., 2003; Kerrest et al., 2009; Voineagu et al., 2009b; Sundararajan et al., 2010), and even short triplet repeats can interfere with nick repair (Pearson et al., 2002). DNA structures, fork stalling, and unreplicated regions of DNA have also been associated with common fragile sites (Zhang and Freudenreich, 2007; Ozeri-Galai et al., 2012). Such hard-to-replicate regions will present a particular challenge to genomic integrity and histone modifications and chromatin-associated factors will be important in maintaining stability of these regions. Additionally, the properties of the repetitive DNA can affect the chromatin structure in the region by forming very stable nucleosomes or excluding nucleosomes (see below). Furthermore, the outcome of a defective repair process can be different in a repetitive DNA sequence compared to non-repeat DNA: the built-in homology surrounding a lesion within a repeat could facilitate repair, but also lead to changes in the repeat number.

CAG repeats form stable DNA hairpins (Mirkin, 2007) and are strong nucleosome positioning elements (Wang et al., 1994; Wang and Griffith, 1995; Volle and Delaney, 2012). Long CAG repeats can activate the checkpoint response (Lahiri et al., 2004; Voineagu et al., 2009a,b; Sundararajan and Freudenreich, 2011), and expansions of the CAG repeat can occur during DNA repair if the process is inefficient (McMurray, 2010). We have found that lesions at an expanded (CAG)_155_ repeat are marked by histone modifications. Both γH2AX and H4 N-terminal tail acetylation at residue K16 are enriched at an expanded CAG repeat during S phase and are required to maintain stability of a (CAG)_85_ repeat during SCR (House et al., 2014), suggesting that these histone modifications are required for high-fidelity repair of structured DNA, potentially through direct recruitment of chromatin remodelers (such as Rsc2) or other repair factors. In human cells, knockdown of class II HDAC9 leads to an increase in CAG repeat expansion frequency (Gannon et al., 2012). However, the opposite is true for HDAC3 and HDAC5, which promote CAG repeat expansions. Though the relevant target for these HDACs is unknown, it was shown that they act within the mismatch repair pathway to protect repeat stability (Gannon et al., 2012).

Whereas CAG repeats preferentially assemble nucleosomes, CGG repeats exclude nucleosomes (Wang, 2007; Kumari and Usdin, 2009). Despite the exclusion of nucleosomes, ATR is required to prevent CGG repeat expansions (Entezam and Usdin, 2008). This suggests that ATR may be phosphorylating H2AX near the CGG repeat to initiate chromatin modifications necessary for DNA repair. Given that CGG repeats are sites of replication fork stalling and chromosomal fragility, it is not surprising that histone modifications associated with DNA damage and repair are found near these sequence elements and are important for repeat stability (Usdin, 2008; Anand et al., 2012; Kumari et al., 2012).

Activation of the checkpoint response by expanded trinucleotide repeats indicates that the structures formed at these sequences are causing damage that initiates a repair event. It is possible that distinct histone modifications are contributing to repair of structured DNA, but the particular combination of modifications that are marking such lesions are only beginning to be identified.

## CHROMATIN MODIFICATIONS IMPORTANT FOR NUCLEOTIDE EXCISION REPAIR (NER)

The nucleotide excision repair pathway is responsible for removing damage that distorts the DNA helix. This type of damage includes UV-induced 6-4photoproducts (6-4PPs) and cyclobutane pyrimidine dimers (CPDs), and repair requires lesion identification and excision. After lesion removal, nucleotides are re-synthesized and the DNA ends are ligated. Chromatin structure must be altered during the NER pathway, both by remodeling and modification of histones, to allow access to the damaged DNA by the proteins participating in the NER repair pathways. Important questions relevant to the NER pathway are whether chromatin relaxation occurs before or after detection of lesions, and the role of histone modifications in chromatin changes versus repair factor recruitment. In addition, chromatin structure must be re-established at the end of the repair process. There are two NER subpathways, and the pathway choice depends on if the DNA damage occurred on a DNA strand that is being actively transcribed: transcription-coupled repair (TCR) repairs damage that occurs on the transcribed strand, whereas global genomic repair (GGR) functions to repair damage that occurs on the nontranscribed strand of active genes or in inactive regions of the genome. Once the damage is recognized and repair is initiated, the two pathways use the same set of repair factors for the downstream events. For a more detailed review of NER in chromatin, see Reed (2011).

### GGR SUBPATHWAY: CHROMATIN MODIFICATIONS INVOLVED IN DAMAGE RECOGNITION AND REPAIR

The GGR subpathway repairs damage that occurs on nontranscribed DNA strands, occurring mostly from exposure to UV radiation. The initial evidence that chromatin modifications occurred during the NER process came from the finding that histones were quickly acetylated after UV irradiation (Ramanathan and Smerdon, 1986). UV irradiation triggers genome-wide histone H3 and H4 hyperacetylation in yeast (Yu et al., 2005). Indeed, acetylation of histone H3, as well as other histone modifications described below, have been shown to facilitate the GGR pathway of NER (**Figure 1E**).

### HISTONE ACETYLATION

After UV irradiation, histone H3-K9 and K14 were shown by ChIP to be hyperacetylated at the repressed *MFA2* promoter in yeast (Yu et al., 2005; **Figure 1E**). This hyperacetylation was dependent on the yeast HAT Gcn5, deletion of which weakened the repair of damage at *MFA2* as assayed by *in vivo* CPD removal (Yu et al., 2005). In yeast, both Gcn5 binding and the resulting histone H3-K9 and K14 acetylation require Rad16 (Teng et al., 2008), a GGR factor with a potential function in chromatin remodeling because it is a SWI/SNF-related family member. Interestingly, the increased Gcn5 binding and H3 acetylation were dependent on both the ATPase and RING domains of Rad16, therefore both translocation and ubiquitin ligase activities are involved (Yu et al., 2011). The resulting H3 acetylation led to a more open chromatin state, measured by restriction enzyme accessibility, which was necessary for GGR (Yu et al., 2011). Recently, another factor in addition to Rad16 has been implicated in enhancing Gcn5 binding after UV damage: the histone H2A variant H2A.Z (Yu et al., 2013). Yeast strains that are deleted for *htz1* are UV sensitive, have reduced histone acetylation, and are defective in removal of DNA damage caused by UV light (Yu et al., 2013). Altogether these studies support the conclusion that UV-induced histone acetylation promotes a more open chromatin structure that is necessary for efficient repair by the NER pathway.

A role for UV-induced histone H3-K9 acetylation during NER has also been observed in human cells. One pathway involves the transcription factor E2F1, which recruits the HAT Gcn5 to UV-damaged DNA (Guo et al., 2011). siRNA-knockdown of Gcn5 impaired recruitment of NER factors XPA and XPC to sites of damage, resulting in less efficient repair of CPDs and 6-4PPs (Guo et al., 2011). A second pathway of histone H3-K9 acetylation during NER has been linked to the function of p53, a tumor suppressor gene. Localization of the HAT p300 to sites of NER was dependent on p53, and H3-K9 acetylation after UV exposure was diminished in p53 mutants (Rubbi and Milner, 2003). Disruption of p300 caused complete NER inhibition, indicating that it is a key HAT in the GGR pathway. By monitoring micrococcal nuclease (MNase) sensitivity, p53 was found to mediate global chromatin relaxation following UV irradiation (Rubbi and Milner, 2003). Therefore, H3 acetylation by both Gcn5 and p300 together may be coordinating chromatin relaxation during NER in human cells.

### HISTONE METHYLATION

Another histone modification recently connected to efficient GGR is histone methylation. Mono and di-methylation of histone H3-K79 was increased in yeast strains with mutations that render the lysines on the H4 N-terminal tail unacetylatable, and the increase in methylation correlated with the severity of UV sensitivity of the H4 K to R mutations (Evans et al., 2008). This finding, therefore, suggests that histone H4 acetylation modulates histone H3-K79 methylation levels during UV damage repair (**Figure 1E**). Histone H3-K79 is methylated by the HMT Dot1, and *dot1Δ* caused sensitivity to UV (Bostelman et al., 2007). Direct evidence for a role for Dot1 and H3-K79me in GGR was obtained by observation of defective repair of CPDs in the non-transcribed strand of *RPB2* in mutants (Tatum and Li, 2011). In contrast, Dot1 and H3-K79 were not necessary for repair in the TCR subpathway, as measured by repair of the transcribed strand of *RPB2* (Tatum and Li, 2011). Therefore, H3-K79 methylation during NER is a GGR-specific modification that may signal for recruitment of the GGR machinery to recognize damage and initiate repair. These findings contrast with a previous study that showed that a H3-K79R yeast mutant displayed almost normal NER at the expressed *RPB2* gene, though NER at the transcriptionally silent cryptic mating-type locus *HML* was impaired (Chaudhuri et al., 2009). However, this study measured NER in both strands of the different loci and did not distinguish between the two strands, which may have therefore missed detection of the repair defect in the nontranscribed strand of *RPB2* observed by Tatum and Li (2011).

In contrast to the increased H3-K79 methylation during GGR observed in yeast, there is a global decrease in trimethylation of a different residue, H3-K9, following UV irradiation in fruit flies (Palomera-Sanchez et al., 2010; **Figure 1E**). UV irradiation increased levels of the histone H3-K9 demethylase, dKDM4B, and H3-K9 demethylation is necessary for repair of the UV lesions as repair of CPDs was impaired in flies with mutated dKDM4B (Palomera-Sanchez et al., 2010). These findings regarding the contrasting role of histone methylation at different H3 residues in the GGR pathway suggest that there may be a specific methylation pattern necessary to signal and recruit factors for repair of UV-induced DNA damage. Intriguingly, Drosophila with p53 mutations had higher levels of trimethylated H3-K9 after UV exposure (Palomera-Sanchez et al., 2010). It would be interesting to determine whether p53 mediates chromatin relaxation in flies, and whether this affects demethylase recruitment as it does HAT recruitment (see above).

### HISTONE UBIQUITINATION

Histone ubiquitination has also been implicated in NER. In human fibroblasts, UV-induced DNA damage resulted in monoubiquitination of H2A-K119, but this modification was not observed in NER-deficient fibroblasts (Bergink et al., 2006; **Figure 1E**). As at DSBs, the NER-induced H2A ubiquitination was dependent on the E2-conjugating enzyme Ubc13 and the ubiquitin E3 ligase RNF8 (Marteijn et al., 2009). Additionally, the UV-damaged DNA-binding protein complex (UV-DDB) contains the subunit DDB2, a ubiquitin E3 ligase that targets histone H2A (Kapetanaki et al., 2006). Ubiquitination of H2A after exposure to UV was shown to be defective in cells from XP group E (XP-E) patients, who have a defect in UV-DDB (Kapetanaki et al., 2006). The ubiquitinated H2A may serve as a recognition signal for damage repair by NER factors that have ubiquitin-binding domains, such as RAD23B, which forms the damage recognition complex with XPC during the initial step of GGR (Kapetanaki et al., 2006). Overall, these findings highlight important associations between histone H2A ubiquitination and the NER pathway.

### HISTONE PHOSPHORYLATION

A chromatin mark that is a hallmark of DSBs, γH2AX is also induced in a NER-dependent manner in UV-exposed non-replicating human cells (O’Driscoll et al., 2003; **Figure 1E**). ATR is the primary kinase for NER-dependent γH2AX (Matsumoto et al., 2007). The precise function of γH2AX in NER remains to be clarified, but if it functions similarly to its role at DSBs, it may be involved in initiating repair events necessary for recruitment of NER factors.

### TCR SUBPATHWAY: POSSIBLE ROLE FOR CHROMATIN MODIFICATIONS

The TCR pathway is activated when RNA polymerase II (RNAPII) stalls at lesions, recruiting factors for repair. Thus some histone modifications associated with active transcription may also have functions in the TCR pathway.

Cockayne syndrome group B (CSB) protein and its homolog Rad26 in yeast are members of the SWI/SNF family of chromatin remodeling enzymes and are important for TCR (Guzder et al., 1996; Selby and Sancar, 1997a). A study by Fousteri et al. (2006) used a co-IP assay to identify proteins associated on the same chromatin fragment after UV treatment. Interactions between CSB, CSA (another TCR factor), and stalled RNAPII were identified, along with the HAT p300 and nucleosome binding protein HMGN1. CSB was necessary for the recruitment of the HAT p300 to stalled RNAPII, whereas the recruitment of HMGN1 was mediated via both CSB and CSA. The interaction between p300 and RNAPII was stimulated by UV and occurred prior to incision of lesions (Fousteri et al., 2006). Given the established role of p300 in NER (see above), it may be that histone acetylation is also needed to facilitate TCR.

In yeast, the association of the TCR factor Rad26 with chromatin is dependent on the presence of elongating RNAPII, and Rad26 is unable to identify lesions in the absence of transcription (Malik et al., 2010). ChIP experiments revealed that histone H3-K36 methylation stimulated the interaction of Rad26 with DNA (Malik et al., 2010). Though not yet tested, the association with Rad26 suggests that H3-K36 methylation may play a role in TCR (**Figure 1E**). However, since Rad26 also promotes transcriptional elongation, it may also be needed more generally to facilitate interaction of Rad26 with chromatin during transcription, rather than having any specific role during TCR.

A connection between ubiquitination and TCR was recently discovered. The deubiquitinating enzyme USP7 is brought to TCR complexes and stabilizes CSB. (Schwertman et al., 2012). TCR factors, including CSB, are known to be ubiquitinated and these could be targets of USP7 activity during TCR, potentially to protect TCR factors from UV-induced degradation. USP7 also deubiquitinates histone H2B and was recently implicated in base excision repair (BER; Khoronenkova et al., 2011). With several possible USP7 targets, the relevant ones for TCR remains to be established.

## CHROMATIN REMODELING IN THE NER PATHWAY

As touched on above, chromatin accessibility plays a key role in NER, and histone acetylation and remodeling may work together to increase access to lesions for repair. Chromatin remodeling during NER has been summarized in a recent review, and compared to remodeling during repair of DSBs by the HR and NHEJ pathways (Lans et al., 2012). The role of chromatin remodelers in the NER subpathways will be highlighted here.

### REMODELING IN THE GGR SUBPATHWAY

In yeast, the GGR factor Rad16 is a SWI/SNF-related family member with ATPase activity (**Table 1**). The ATPase activity of Rad16 is required for efficient repair (Ramsey et al., 2004; Yu et al., 2011), and it is therefore assumed that Rad16 is acting as a chromatin remodeler, although nucleosome displacement by Rad16 has not been directly observed. In addition, Rad16 has been shown to promote Gcn5-dependent histone H3 acetylation during the repair of UV damage, and this leads to increased chromatin accessibility that is necessary for efficient damage repair (Yu et al., 2011).

A link between SWI/SNF chromatin remodeling and NER was discovered in yeast and is now well established. The NER damage recognition complex consisting of Rad4 and Rad23 (yeast homolog of human XPC-RAD23B) co-purified with Snf6 and Snf5, both SWI/SNF chromatin remodeling complex subunits, and the interactions increased with UV exposure (Gong et al., 2006; **Table 1**). Inactivation of SWI/SNF via *snf6Δ* reduced restriction enzyme accessibility and affected the rate of CPD removal at the silent *HML* locus, implying that SWI/SNF is remodeling during NER (Gong et al., 2006). The double mutant *rad16Δ snf6Δ* was more UV sensitive than the *rad16Δ* single mutant, suggesting that Snf6 may have a role in TCR as well as GGR (Gong et al., 2006). Since Snf6 interacts with Rad4–Rad23 and Rad4 functions in both NER pathways (Verhage et al., 1994), it is possible for Snf6 to influence repair by both GGR and TCR. Additionally, following UV irradiation, chromatin was remodeled to increase DNA accessibility at *MFA2*, measured by restriction enzyme accessibility, which was partially dependent on the activity of the SWI/SNF ATPase Snf2 (Yu et al., 2005; **Table 1**). Overall, these findings support a function for SWI/SNF remodeling in the NER pathway.

Evidence for SWI/SNF chromatin remodeling during NER in mammals comes from BRG1 knockdown experiments that showed reduced repair of CPDs following damage with UV radiation, whereas restoring BRG1 in cells lacking the endogenous protein showed stimulation of NER (Zhang et al., 2009; Zhao et al., 2009; **Table 1**). In addition, SWI/SNF subunits BRG1 and SNF5 have been shown to physically interact with XPC (Ray et al., 2009; Zhao et al., 2009). In *C. elegans*, orthologs of mammalian SWI/SNF, including BRG1, BRM/SMARCA2, SNF5, PBRM1, and BAF155/SMARCC1 were implicated in survival after UV exposure (Lans et al., 2010).

A recent study revealed a function for poly (ADP-ribosyl)ation and chromatin remodeling during NER repair. Immunoprecipitation of DDB2 complexes and subsequent mass spectrometry analysis of the interacting proteins identified PARP1 as a DDB2-associated factor in human cells (Pines et al., 2012). This interaction was dependent on UV irradiation and promoted the synthesis of poly(ADP-ribose; PAR) chains in chromatin with UV-induced lesions. In DDB2-deficient cells, there was substantially less PAR immunofluorescence at UV damaged sites compared to wild-type (Pines et al., 2012). The poly(ADP-ribosyl)ation recruited the SWI/SNF chromatin remodeler ALC1 to sites of UV-induced DNA lesions. Knockdown of ALC1 using shRNA resulted in UV-sensitive cells that had deficient repair of CPDs and 6-4PPs, indicating that ALC1 activity is critical to the GGR/NER pathway (Pines et al., 2012; **Table 1**).

Chromatin remodeling by INO80 is also implicated in NER (**Table 1**). Yeast *ino80Δ* mutants and mammalian cells with RNAi knockdown of Ino80 are UV sensitive (Shen et al., 2000; Wu et al., 2007). In yeast, UV-induced recruitment of Ino80 to chromatin occurs through interactions with the Rad4–Rad23 NER damage recognition complex (Sarkar et al., 2010). In mammals, Ino80 is recruited to UV-damaged chromatin, and deletion of two INO80 complex subunits, INO80 and ARP5, resulted in defective repair of UV lesions (Jiang et al., 2010). In addition, INO80-deficient cells failed to recruit the NER factors XPC and XPA, suggesting that INO80 chromatin remodeling may be necessary for lesion recognition and incision (Jiang et al., 2010). The links between Ino80 and NER in both yeast and mammalian systems, the UV repair defects, and the direct interactions with NER factors all support the conclusion that Ino80 is another chromatin remodeler with an important role in the NER pathway.

### REMODELING IN THE TCR SUBPATHWAY

Cockayne syndrome group B and its homolog Rad26 in yeast are DNA-dependent ATPases of the SWI/SNF family of ATP-dependent chromatin remodeling enzymes acting in the TCR pathway (Guzder et al., 1996; Selby and Sancar, 1997a). Both CSB and Rad26 have been shown to affect chromatin structure, based on *in vitro* experiments for CSB, and mutant phenotypes for Rad26 (Citterio et al., 2000; Gregory and Sweder, 2001; **Table 1**). In addition, both CSB and Rad26 enhance transcriptional elongation (Selby and Sancar, 1997b; Lee et al., 2001, 2002). CSB has been shown to be necessary for recruitment of repair factors to sites of damage repaired by the TCR pathway (Fousteri et al., 2006). Recently, Rad26 was found to promote ejection of the H2A-H2B dimer during transcription of the *GAL1* gene (Malik and Bhaumik, 2012). This regulation of chromatin structure by Rad26 is critical for transcription and may be necessary for recruitment of repair factors during TCR to allow access to the DNA lesions. Future studies should directly address whether the role of Rad26 in promoting H2A-H2B dimer eviction also contributes to efficient TCR.

There are some suggestions of ISWI chromatin remodeling in NER and/or TCR. In experiments using synthetic dinucleosomes containing 6-4PPs, recombinant Drosophila ACF stimulated lesion excision (Ura et al., 2001; **Table 1**). Also, knockdown of ISWI in human cells and *C. elegans* results in a modest UV sensitive phenotype (Lan et al., 2010; Lans et al., 2010; Sanchez-Molina et al., 2011). Additionally, the human ISWI isoform SMARCA5 is recruited to UV-induced DNA lesions, where it promotes binding of the TCR factor CSB and restart of damage-stalled transcription (Aydin et al., 2014). Intriguingly, purification of the human WICH complex (WSTF-SNF2H), an ISWI family complex, identified an interaction with CSB that was confirmed by co-immunoprecipitation (Cavellan et al., 2006), adding another link between ISWI and TCR. Thus, ISWI remodeling may work together with Rad26/CSB to facilitate lesion repair during transcription.

## BASE EXCISION REPAIR (BER) WITHIN CHROMATIN

DNA bases damaged by, for example, oxidation and alkylation, are repaired through BER. The damage repaired by BER does not significantly distort the DNA and therefore does not stall the replication or transcription machinery. The BER pathway is initiated when a glycosylase enzyme recognizes and excises the damaged base, leaving an abasic site. The abasic site is processed by apyrimidinic/apurinic endonuclease (APE), which cleaves the phosphodiester backbone, leaving a base gap. Then, DNA polymerase inserts the missing base(s) and DNA ligase seals the nick, completing the BER repair process. Although the role of chromatin structure in the BER pathway has not been investigated in depth, some links to histone modification and remodeling have been identified.

There is some recent evidence for the importance of histone modifications during BER. USP7 is a ubiquitin-specific human protease which deubiquitinates histone H2B *in vitro*, though it also targets non-histone substrates that include p53 (Li et al., 2002). Upon siRNA knockdown of USP7, the levels and activity of BER enzymes were not changed, but the accessibility of DNA and the repair rate of oxidative lesions were both reduced (Khoronenkova et al., 2011). These results support their model for H2B ubiquitination state affecting BER, though it will be important to address whether histone H2B, or another protein substrate, is the relevant *in vivo* target during BER. A connection between acetylation and the BER pathway was observed in mammalian cells by co-immunoprecipitation and co-localization of thymine DNA glycosylase (TDG) and the HAT p300 (Tini et al., 2002). P300/TDG complexes are competent for histone acetylation and TDG itself is also acetylated by p300, therefore TDG may be the relevant target of p300 (Tini et al., 2002). To date, no other histone modifications have been demonstrated to affect BER and therefore this is an interesting area for further study.

Multiple *in vitro* studies have investigated whether BER enzymes can function properly in the context of a nucleosome-containing template. Using uracil-containing oligonucleosome arrays, the activities of uracil DNA glycosylase (UDG), which recognizes uracil in DNA, and APE, which recognizes abasic sites, were both uninhibited, suggesting that the initial steps of BER by UDG and APE can act efficiently in intact chromatin (Nakanishi et al., 2007). However, synthesis by the polymerase functioning in BER, DNA polymerase β, was significantly reduced in the oligonucleosome array (Nakanishi et al., 2007). This inhibition was lessened upon addition of purified yeast Isw1 and Isw2, both chromatin remodelers of the ISWI family, suggesting that remodeling could be crucial for later repair events in BER within compact chromatin (Nakanishi et al., 2007; **Table 1**). The *in vitro* mechanism of BER has also been studied using an 8-oxo-7, 8-dihydroguanine (8-oxoG) lesion on reconstituted nucleosomes. Activities of murine 8-oxoguanine DNA glycosylase (OGG1), human APE, and human polymerase β were all reduced compared to their activity on a naked DNA substrate (Menoni et al., 2007). The addition of the yeast SWI/SNF complex stimulated the activity of all three BER enzymes in the repair of the oxidative lesion in the nucleosomal array, to a level comparable to their activity on naked DNA (**Table 1**). This effect required SWI2/SNF2 dependent remodeling but not relocation of nucleosomes (Menoni et al., 2007). These two *in vitro* studies utilized different types of BER lesions in the context of a nucleosome substrate. Both concluded that chromatin remodeling promotes polymerase β activity, however, differences were seen for the activities of the glycosylases (UDG, OGG1) and endonuclease APE on the nucleosome substrates that were used, which may be related to the lesion type or the nucleosome substrate itself. Overall, these studies both point to a role for the SWI/SNF family remodelers for efficient BER, though an *in vivo* role must still be established. Recently, it was shown that depletion of STH1, the ATPase subunit of RSC, results in sensitivity to MMS, and BER is considerably inhibited in cells lacking STH1 (Czaja et al., 2014). This establishes the first *in vivo* link between chromatin remodeling and BER.

## HISTONE MODIFICATIONS AND NUCLEOSOME REMODELING DURING MISMATCH REPAIR (MMR)

Postreplication mismatch repair (MMR) is initiated when a base mismatch escapes the DNA polymerase proofreading machinery. In human cells, MMR is regulated by histone H3-K36 trimethylation (**Figure 1F**). H3-K36me3 is required *in vivo* to recruit the heterodimer MSH2-MSH6 (MutSα) to chromatin through the Pro-Trp-Trp-Pro (PWWP) domain of MSH6, a domain that specifically interacts with H3-K36me3 (Li et al., 2013). Since H3-K36me3 is abundant during G1 and early S phase, it is thought that this ensures the enrichment of MutSα on DNA during the period when mismatches are likely to arise. Cells that lack the H3-K36 trimethyltransferase SETD2 have altered MSH6 localization and a mutator phenotype, but are not defective in MMR *in vitro* (Li et al., 2013). Whether additional histone modifications are involved in MMR is as yet unknown; good candidates may be those associated with the progression of DNA replication, such as H3-K56 (Kadyrova et al., 2013).

Because nucleosomes become disassembled in front of a replication fork, newly replicated DNA is relatively nucleosome-poor and MMR may not need robust chromatin remodeling to effectively compete with nucleosomes. However, fully formed nucleosomes have been observed about 250 bp from a replication fork, and there are intermediates in the assembly process in the region in between (Sogo et al., 1986; Jackson, 1988). Therefore, the MMR machinery is likely to encounter some completely formed nucleosomes in addition to nucleosome intermediates. There is some evidence for interaction between MMR factors and the histone H3–H4 chaperone chromatin assembly factor 1 (CAF-1). Mismatch correction reactions with HeLa cell extracts demonstrated that replication error correction occurs on DNA that is packaged into nucleosomes by CAF-1 (Kadyrova et al., 2011). However, in a combined *in vitro* MMR and nucleosome assembly assay, a mismatch in a nicked plasmid substrate delayed loading of nucleosomes in a human cell extract (Schöpf et al., 2012), suggesting that MMR interferes with nucleosome assembly. The balance between MMR and chromatin reassembly may be regulated by a physical interaction between MutSα, specifically MSH6, and the p150 subunit of CAF-1 (Schöpf et al., 2012).

In addition to these interactions between MMR and chromatin assembly factors, passive chromatin remodeling assists the MMR process. Using *in vitro* experiments with reconstituted nucleosomes and purified human proteins, the MMR initiation heterodimer MutSα disassembles nucleosomes (Javaid et al., 2009). Nucleosome remodeling by MutSα required a mismatch and translocation of the complex as a sliding clamp along the DNA (Javaid et al., 2009). The nucleosome remodeling function required ATP binding but not hydrolysis, suggesting that the remodeling function is passive. Histone H3 acetylation or an H3 acetylation mimic (H3-K115Ac, H3-K122Ac, H3-K56Q), enhanced the remodeling function of MutSα (Javaid et al., 2009). Additionally, phosphorylation of histone H3-T118 enhanced nucleosome disassembly by MutSα by 25-fold *in vitro* (North et al., 2011). However, no *in vivo* investigation has been done yet to support a role for H3 phosphorylation or acetylation in MMR. There is also evidence that passive MutSα-dependent nucleosome disassembly may not be sufficient, as human MutSα bound poorly to a substrate with a mismatch within a nucleosome (Li et al., 2009). In addition, nucleosomes blocked ATP-induced sliding of MutSα along the DNA when there was a mismatch between two nucleosomes (Li et al., 2009). Overall, these findings indicate that nucleosomes likely inhibit the MMR process to some degree, and active remodeling may yet be found to play a role in MMR.

## CONCLUDING REMARKS

The interplay between histone modifications and DNA repair likely creates a diverse array of cellular responses to DNA damage based on the type of lesion and the preferred pathway of repair for a particular lesion. γH2AX is the first detectable histone modification in response to DSBs, but it appears to be a general initial modification, acting as a broad signal of DNA damage, activating signaling cascades in response to stalled forks, gaps, DNA structures, and UV lesions, as well as DSBs. The subsequent, downstream histone modifications may guide repair to the appropriate pathway based on lesion type.

A major unanswered question for many of the histone modifications summarized here is their mechanism of action. Do histone marks recruit specific repair factors or remodelers via direct interaction, or change local chromatin accessibility in a more general way, or a combination of both? Several examples of direct interactions exist, for example Arp4, a subunit of INO80, SWR1, and NuA4 complexes, binds specifically to yeast H2A phosphorylated at Ser129 (Downs et al., 2004). In mammalian cells, the repair mediator MDC1 binds directly to γH2AX via tandem BRCT domains (Uziel et al., 2003; Lukas et al., 2004; Stucki et al., 2005). In addition, other roles for modifications can also be envisioned, such as repositioning of the damaged area to another nuclear compartment to direct appropriate repair (Dion and Gasser, 2013).

Specific combinations of histone modifications may also be important to differentially favor the recruitment of particular repair factors. Depending on the interaction of the repair proteins with the histone modifications, progressive histone modifications after DNA damage could influence repair pathway choice or progression. One example of this during DSB repair in human cells is Tyr142 on H2AX, which is phosphorylated in the absence of DNA damage by the WSTF kinase (Xiao et al., 2009). However, upon DNA damage and phosphorylation of H2AX at Ser139, Tyr142 is dephosphorylated by the Eya1 and Eya3 tyrosine phosphatases (Cook et al., 2009). While the di-phospho γH2AX can be bound by the repair factor MCPH1, MDC1 only efficiently binds γH2AX once it is dephosphorylated at Tyr142 (Singh et al., 2012), thus directing binding of repair factors in an orderly fashion. In most cases, relatively little is understood about the order of the occurrence of the modifications depicted in **Figure 1**, and whether some work together to recruit factors, change chromatin structure, or signal completion of repair.

It is reasonable to expect that different lesions will also require a different chromatin environment to promote repair, and thus unique levels and types of chromatin remodeling. End resection, D-loop extension during HR, gap filling, fork restart, and repair of base lesions or mismatches could each require a certain degree of nucleosome movement or displacement. For instance, if a homologous template is utilized for repair, chromatin remodeling will require the movement of several nucleosomes at the targeted, homologous sequence to allow invasion into the template sequence and subsequent copying, as well as at the site of the lesion if any resection is required for repair; on the other hand, repair of base lesions or gap filling without strand invasion may not require as substantial of a remodeling process. Finally, repair resolution will require reestablishment of the chromatin state and DNA damage checkpoint recovery. Depending on the chromatin modifications that took place during repair, the disruption to the chromatin will vary and thus may require different factors to reestablish the normal chromatin state.

Some combinations of histone modifications that distinguish repair pathways from one another are summarized here, but many remain to be identified. Understanding how these histone modifications work together to contribute to repair will further our understanding of how the DNA repair machinery functions within the context of the chromatin structure. Additionally, roles for chromatin modifications in designating repair choice, orderly progression of repair, turnover of repair factors, and resolution of the damage response may be revealed.

## Conflict of Interest Statement

The authors declare that the research was conducted in the absence of any commercial or financial relationships that could be construed as a potential conflict of interest.
